# The calibrated model-based concordance improved assessment of discriminative ability in patient clusters of limited sample size

**DOI:** 10.1186/s41512-019-0055-8

**Published:** 2019-06-06

**Authors:** David van Klaveren, Ewout W. Steyerberg, Mithat Gönen, Yvonne Vergouwe

**Affiliations:** 1 Department of Public Health, Erasmus University Medical Center, Rotterdam, The Netherlands; 2 Predictive Analytics and Comparative Effectiveness Center, Institute for Clinical Research and Health Policy Studies, Tufts Medical Center, Boston, USA; 3 Department of Biomedical Data Sciences, Leiden University Medical Center, Leiden, The Netherlands; 4 Epidemiology and Biostatistics, Memorial Sloan Kettering Cancer Center, New York, USA

**Keywords:** Prediction model, Model performance, Discrimination, Concordance, Clustered data, Multilevel regression, Traumatic brain injury

## Abstract

**Background:**

Discriminative ability is an important aspect of prediction model performance, but challenging to assess in clustered (e.g., multicenter) data. Concordance (c)-indexes may be too extreme within small clusters. We aimed to define a new approach for the assessment of discriminative ability in clustered data.

**Methods:**

We assessed discriminative ability of a prediction model for the binary outcome mortality after traumatic brain injury within centers of the CRASH trial. With multilevel logistic regression analysis, we estimated cluster-specific calibration slopes which we used to obtain the recently proposed calibrated model-based concordance (*c-mbc*) within each cluster*.* We compared the *c-mbc* with the naïve c-index in centers of the CRASH trial and in simulations of clusters with varying calibration slopes.

**Results:**

The *c-mbc* was less extreme in distribution than the c-index in 19 European centers (internal validation; *n* = 1716) and 36 non-European centers (external validation; *n* = 3135) of the CRASH trial. In simulations, the *c-mbc* was biased but less variable than the naïve c-index, resulting in lower root mean squared errors.

**Conclusions:**

The *c-mbc*, based on multilevel regression analysis of the calibration slope, is an attractive alternative to the c-index as a measure of discriminative ability in multicenter studies with patient clusters of limited sample size.

**Electronic supplementary material:**

The online version of this article (10.1186/s41512-019-0055-8) contains supplementary material, which is available to authorized users.

## Background

Assessing the performance of a prediction model is of great practical importance [1, 2]. An essential aspect of model performance is separating subjects with good outcome from subjects with poor outcome (discrimination) [3]. Harrell’s concordance-index (c-index) is often used to assess discrimination [4]. The c-index estimates the probability that for two randomly chosen subjects with different outcomes, the model predicts a higher risk for the subject with poorer outcome (concordance probability). In addition to the c-index, we recently introduced a model-based concordance measure (*mbc*), similar to the concordance probability estimator proposed for proportional hazards regression models by Gönen and Heller [5, 6]. The *mbc* is the expected concordance probability of a regression model under the assumption that the regression model is “valid”, i.e., outcomes are generated according to this regression model. The *mbc* at external validation is the closed form variant of the previously proposed case-mix corrected c-index [7]. The difference between the *mbc* at model development and the *mbc* at external validation indicates the change in discriminative ability attributable to the difference in case-mix heterogeneity between the development and validation data. The calibrated *mbc* (*c-mbc*)—based on predictions recalibrated to the external validation data—also takes (in)validity of the regression coefficients, including the intercept, into account when measuring the discriminative ability in external data.

In risk modeling, patient data is often clustered. A typical example is multicenter patient data, i.e., data of patients who are treated in different centers. We have suggested summarizing the discriminative ability with random-effects meta-analysis of the cluster-specific c-index, because the discriminative ability often varies between clusters of patients [8]. However, for small clusters, the cluster-specific c-index may be too extreme. Extreme estimates are also a problem for cluster-specific calibration intercepts and slopes. Multilevel regression analysis can provide less extreme (“shrunk”) random effect estimates, trading off variance with bias [9–11]. The random effect estimates of calibration intercepts and slopes can also be used for calculation of the *c-mbc*, which is the expected concordance probability under the assumption that the random effect estimates of the calibration intercept and slope are valid, i.e., outcomes are generated according to the calibrated regression model. Similar to the cluster-specific random intercept and slope estimates, we may expect the cluster-specific *c-mbc* to be more stable than the c-index.

We aimed to study this new approach for assessment of discriminative ability in clustered data, especially for small clusters. We compare the cluster-specific *c-mbc*—based on random effect estimates of calibration intercepts and slopes—with the naïve cluster-specific c-index in a case study with substantial variation in calibration slopes across small clusters. We study the trade-off between variance and bias of the cluster-specific c-index and *c-mbc* in a simulation study.

## Methods

### The (calibrated) model-based concordance

The recently proposed *mbc* (equations in Appendix) estimates a logistic or proportional hazards regression model’s concordance probability at apparent validation [6]. The *mbc* is asymptotically equivalent to the c-index, with exact equality when the model contains only one categorical predictor. This *mbc* is a function of the regression coefficients and the covariate distribution and does not use observed outcomes. Consequently, in an external validation population, the *mbc* is not influenced by the validity of the regression coefficients and merely assesses the expected discriminative ability of the model, similar to a previously proposed case-mix corrected c-index [10]. To assess the influence of overall regression coefficient validity on the concordance probability, we first estimate the calibration intercept *γ*_0_ and the calibration slope *γ*_1_ in the validation data, i.e., the regression coefficients of a model that regresses the observed outcomes on the linear predictors *Xβ* in the validation data [12]. If ${\widehat{\gamma}}_1=1$, the regression coefficients are on average valid in the validation data. In contrast, ${\widehat{\gamma}}_1<1$ indicates a weaker association between the linear predictor and the outcomes in the validation data. The $mbc\left({\widehat{\gamma}}_0+{\widehat{\gamma}}_1 X\beta \right)$, which we label calibrated model-based concordance (*c-mbc*), incorporates both the influence of case-mix heterogeneity and the overall validity of the regression coefficients *β* on the discriminative ability of the prediction model. Variance estimates of the *mbc* and the *c-mbc* in model development and external validation settings are easily available as well [6]*.*

### The calibrated model-based concordance in clustered data

When data is clustered, we denote with *x*_*ik*_ the baseline characteristics vector for patient *i* in cluster *k*, and with ${z}_{ik}={x}_{ik}^T\beta$ the corresponding linear predictors of a logistic regression model with regression coefficients *β* and intercept *β*_0_. We can incorporate calibration intercepts *γ*_0*k*_ and slopes *γ*_1*k*_ for individual clusters in a multilevel logistic regression model [9]:

1 $$ {\displaystyle \begin{array}{l}\mathrm{logit}\left({p}_{ik}\right)=\mathrm{offset}\left({\beta}_0\right)+{\gamma}_{0k}+{\gamma}_{1k}{z}_{ik}\\ {}{\gamma}_k=\left[\begin{array}{c}{\gamma}_{0k}\\ {}{\gamma}_{1k}\end{array}\right]\sim N\left(\left[\begin{array}{c}{\gamma}_0\\ {}{\gamma}_1\end{array}\right],\left[\begin{array}{cc}{\sigma}_0^2& {\rho \sigma}_0{\sigma}_1\\ {}{\rho \sigma}_0{\sigma}_1& {\sigma}_1^2\end{array}\right]\right)\end{array}} $$

The best linear unbiased predictors ${\widehat{\gamma}}_{0k}$ and ${\widehat{\gamma}}_{1k}$ represent random effect estimates of the calibration intercept and the calibration slope in cluster *k*. Although the naming and interpretation of ${\widehat{\gamma}}_{0k}$ and ${\widehat{\gamma}}_{1k}$ has been debated, we will loosely call them random effect estimates—accompanied by confidence intervals—because we will repeatedly compare them with fixed effect estimates [13, 14]. The random effects estimates of the intercept and slope in cluster *k* can be plugged into Eq. 7. With *Z*_*k*_ = *X*_*k*_*β* the linear predictors of patients in cluster *k*, we obtain the *c-mbc* of a multilevel logistic regression model in cluster *k*:

2 $$ c-{mbc}_k= mbc\left({\beta}_0+{\hat{\gamma}}_{0k}+{\hat{\gamma}}_{1k}{Z}_k\right) $$

## Results

### Case study of traumatic brain injury

#### Case study design

We present a case study of predicting mortality after traumatic brain injury (TBI). We used patients enrolled in the Medical Research Council Corticosteroid Randomisation after Significant Head Injury trial (registration ISRCTN74459797), who were recruited between 1999 and 2004 [15]. This was a large international double-blind, randomized placebo-controlled trial of the effect of early administration of a 48-h infusion of methylprednisolone on outcome after head injury. We considered patients with moderate or severe brain injury (GCS total score ≤ 12) and observed 6-month Glasgow Outcome Scale (GOS) [16, 17]. Patients (*n* = 1716) who were treated in one of 19 European centers with more than 10 patients experiencing the event were included in the analysis. A logistic regression model was fitted—ignoring clustering—with age, GCS motor score and pupil reactivity as covariates, similar to previously developed risk models [18, 19]. To assess the performance of this model’s linear predictors within each cluster, we estimated the cluster-specific calibration intercept, calibration slope, and c-index. We compared the estimates with random effect estimates of the calibration intercept and slope (multilevel logistic regression model in Eq. 1) and the *c-mbc* (Eq. 2), respectively. All the analyses were done in R software, and multilevel regression analysis was done with the lme4 package [20, 21].

#### Case study results

**Fig. 1:**
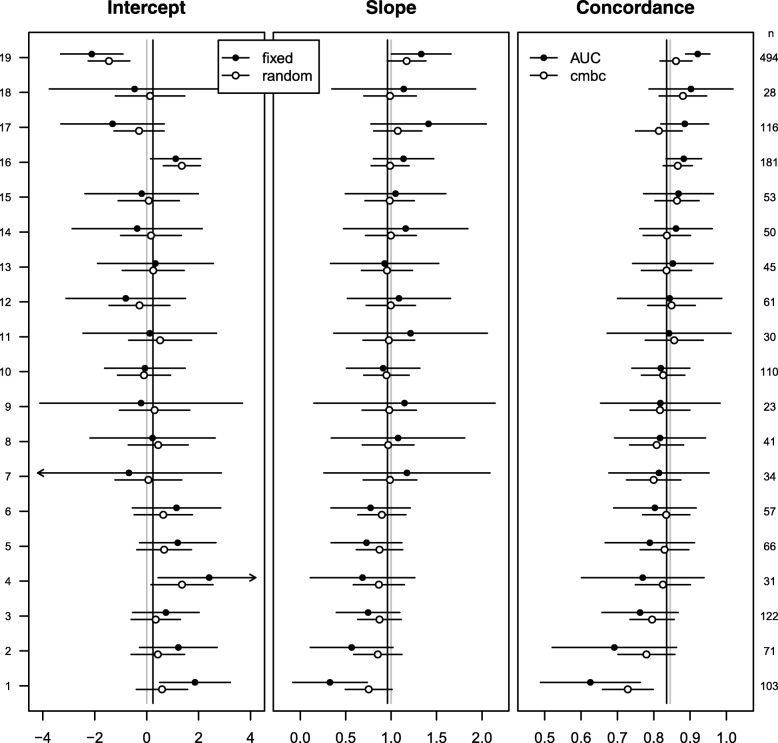
Performance measures at internal validation across 19 centers of the CRASH trial. Closed dots represent fixed effect intercept estimates; fixed effect slope estimates; and c-indexes in the first, second, and third panel, respectively. Open dots represent random effects intercept estimates, random effects slope estimates, and calibrated model-based concordance estimates in the first, second, and third panel, respectively. Gray vertical lines represent effect estimates of intercept (0) and slope (1) in the original regression model, together with the expected pooled concordance (0.85). Black vertical lines represent fixed effect estimates of intercept (0.24) and slope (0.96) in a multilevel regression model, together with the expected pooled concordance (0.84). The number of patients in each center is indicated on the right *y*-axis

At internal validation, we found substantial heterogeneity in calibration intercepts and slopes (*σ*_0_ = 0.82; *σ*_1_ = 0.16; *ρ* =  − 0.76). The mean of the cluster-level calibration intercepts (*γ*_0_ = 0.24) and the mean of the cluster-level calibration slopes (*γ*_1_ = 0.96) were close to the apparent estimates of the calibration intercept (≡ 0) and the calibration slope (≡ 1). As expected, random effects estimates of the calibration intercept and slope were less heterogeneous and had narrower 95% confidence intervals than fixed effect estimates (left and middle panels of Fig. 1; Additional file 1: Table S1). Similarly, the *c-mbc* based on random effect estimates was less heterogeneous and had narrower 95% confidence intervals than the cluster-specific c-index (right panel of Fig. 1).

**Fig. 2:**
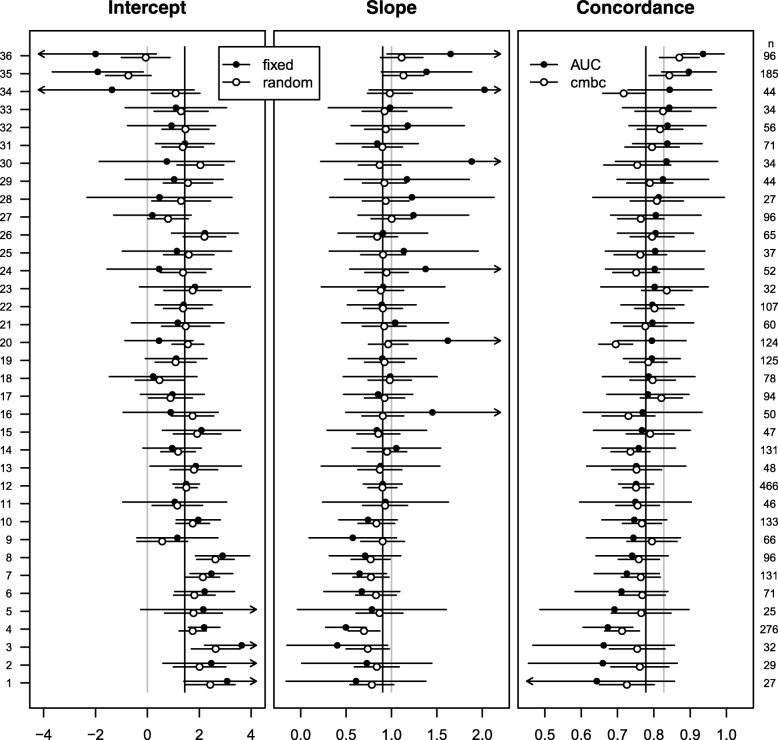
Performance measures at external validation across 36 centers of the CRASH trial. Closed dots represent fixed effect intercept estimates; fixed effect slope estimates; and c-indexes in the first, second, and third panel, respectively. Open dots represent random effects intercept estimates, random effects slope estimates, and calibrated model-based concordance estimates in the first, second, and third panel, respectively. Gray vertical lines represent effect estimates of intercept (0) and slope (1) in the original regression model, together with the expected pooled concordance (0.83). Black vertical lines represent fixed effect estimates of intercept (1.44) and slope (0.90) in a multilevel regression model, together with the expected pooled concordance (0.78). The number of patients in each center is indicated on the right *y*-axis

At external validation, for patients who were treated in one of 36 non-European centers with more than 10 patients experiencing the event (*n* = 3135), the intercept was poorly calibrated (*γ*_0_ = 1.44) and the linear predictors slightly overfitted (*γ*_1_ = 0.90). The heterogeneity in the calibration intercept and slope was very similar to the European setting (*σ*_0_ = 0.81; *σ*_1_ = 0.15; *ρ* =  − 0.79). Differences between fixed effect estimates and random effects estimates and between the c-index and the *c-mbc* were comparable to the European setting (Fig. 2; Additional file 1: Table S2).

### Simulation study

#### Design of the simulation study

To study the trade-off between variance and bias of the cluster-specific c-index and the *c-mbc*, we simulated validation studies of a logistic regression model in 40 clusters of 200 patients. To incorporate heterogeneity in true intercepts and slopes across clusters, we drew once for each cluster *k* a true calibration intercept *γ*_0*k*_ and a true calibration slope *γ*_1*k*_ from independent normal distributions with means *γ*_0_ = 0 and *γ*_1_ = 1, respectively, and standard deviations *σ*_0_ = *σ*_1_ = 0.2.

In each of 2000 replications, we generated for patient *i* in cluster *k* a continuous baseline linear predictor *z*_*ik*_ from a standard normal distribution and a binary outcome from a Bernoulli distribution with success probability [1 + exp {−(−2 + *γ*_0*k*_ + *γ*_1*k*_*z*_*ik*_)}]^−1^. With such an average intercept of − 2, the expected event rate in a typical cluster (*γ*_0*k*_ = 0; *γ*_1*k*_ = 1) is 15.5%. We produced cluster-specific (fixed effect) estimates of the calibration intercept and slope and the cluster-specific c-index in each replication. Furthermore, we produced random effect estimates of the calibration intercept and slope (multilevel logistic regression model of Eq. 1) and the *c-mbc* (Eq. 2) in each replication.

We summarized the cluster-specific estimates of the calibration intercept, the calibration slope and the concordance probability with the average deviation from the true value (bias), the standard deviation (square root of the variance), and the root of the average squared difference with the true values (root mean squared error [rmse]). To obtain the true concordance probability within each cluster *k*, we used *mbc*(−2 + *γ*_0*k*_ + *γ*_1*k*_*Z*_*k*_), with *Z*_*k*_ the vector of linear predictors for patients in cluster *k*, because it is equal to the mean c-index in infinitely many replications of cluster *k* assuming that *γ*_0*k*_ and *γ*_1*k*_ are true [6].

#### Main results of the simulation study

**Fig. 3:**
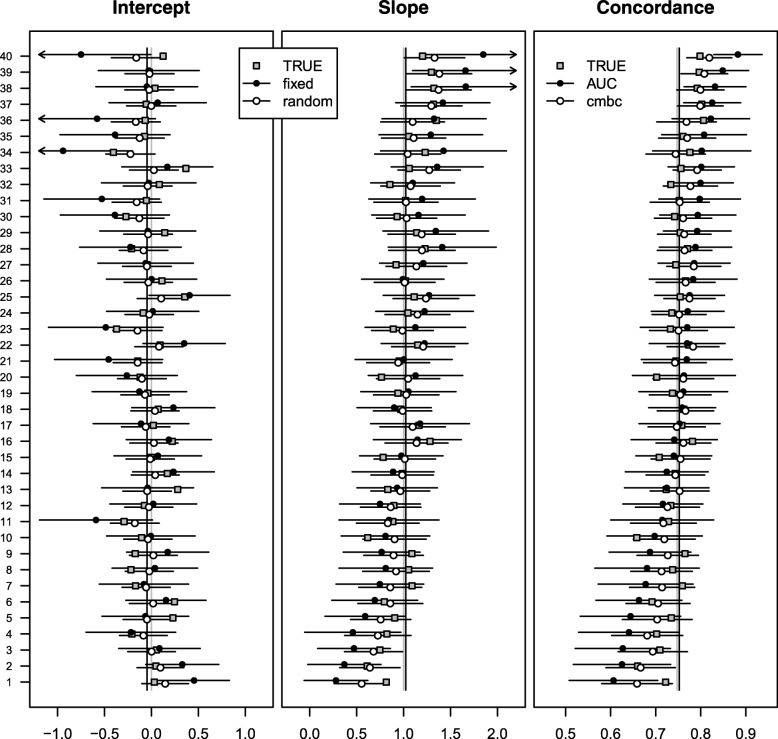
Performance measures across 40 centers of 200 simulated patients. Gray squares represent true values of intercept, slope, and concordance probability. Closed dots represent fixed effect intercept estimates; fixed effect slope estimates; and c-indexes in the first, second, and third panel, respectively. Open dots represent random effects intercept estimates; random effects slope estimates; and calibrated model-based concordance estimates in the first, second, and third panel, respectively. Gray vertical lines represent effect estimates of intercept and slope in the original regression model, together with the expected pooled concordance. Black vertical lines represent fixed effect estimates of intercept and slope in a multilevel regression model, together with the expected pooled concordance

The cluster-specific c-index was unbiased (Table 1). The bias of the *c-mbc* increased with the deviation of the true cluster-specific concordance probability from the overall average. Due to a positive trade-off with variance (lower standard deviation), the rmse of the *c-mbc* was generally lower than the rmse of the c-index. Similar plots as for the case study (Figs. 1 and 2) could be drawn for each replication of the simulation study. We plotted the estimates from the first replication, including true cluster-specific values (Fig. 3). Again, random effects estimates of calibration intercept and slope and the *c-mbc* were less heterogeneous and had narrower 95% confidence intervals than fixed effect estimates and the c-index, respectively.

**Table 1:** Simulation characteristics (2000 replications) of c-index and *c-mbc* across 40 centers of 200 patients

Cluster	True	Bias		SD		rmse	
	Concordance	C-index	*c-mbc*	C-index	*c-mbc*	C-index	*c-mbc*
1	0.664	0.002	0.051	0.055	0.027	0.055	0.057
2	0.666	0.001	0.047	0.059	0.027	0.059	0.054
3	0.695	0.001	0.032	0.053	0.024	0.052	0.040
4	0.699	0.000	0.025	0.056	0.024	0.055	0.034
5	0.702	0.000	0.026	0.054	0.024	0.053	0.034
6	0.705	− 0.001	0.030	0.049	0.025	0.048	0.038
7	0.710	− 0.002	0.022	0.051	0.023	0.050	0.031
8	0.712	− 0.001	0.015	0.056	0.024	0.055	0.027
9	0.712	0.001	0.028	0.048	0.024	0.047	0.035
10	0.718	0.002	0.020	0.051	0.023	0.050	0.029
11	0.726	0.001	0.004	0.056	0.024	0.055	0.022
12	0.727	− 0.001	0.001	0.058	0.024	0.057	0.022
13	0.727	0.000	0.017	0.047	0.023	0.046	0.027
14	0.727	0.001	0.010	0.051	0.023	0.050	0.023
15	0.731	0.001	0.008	0.049	0.022	0.048	0.021
16	0.735	− 0.001	− 0.001	0.053	0.023	0.053	0.021
17	0.735	0.001	0.006	0.050	0.022	0.049	0.021
18	0.738	0.000	0.001	0.052	0.023	0.051	0.020
19	0.741	0.001	0.006	0.047	0.022	0.046	0.020
20	0.744	0.001	0.007	0.045	0.022	0.043	0.020
21	0.750	0.000	0.002	0.046	0.021	0.045	0.019
22	0.751	0.001	− 0.004	0.049	0.022	0.047	0.019
23	0.755	0.001	0.006	0.043	0.023	0.041	0.021
24	0.755	0.000	− 0.008	0.049	0.022	0.048	0.020
25	0.757	0.002	− 0.007	0.046	0.021	0.045	0.020
26	0.757	0.002	− 0.013	0.051	0.022	0.049	0.023
27	0.763	− 0.002	− 0.015	0.050	0.022	0.049	0.024
28	0.763	0.000	− 0.014	0.048	0.021	0.047	0.023
29	0.765	0.000	0.000	0.041	0.022	0.040	0.019
30	0.770	0.001	− 0.009	0.043	0.022	0.042	0.020
31	0.772	0.002	− 0.011	0.043	0.021	0.041	0.021
32	0.774	0.001	− 0.015	0.044	0.021	0.042	0.023
33	0.780	0.000	− 0.015	0.042	0.021	0.041	0.024
34	0.787	− 0.001	− 0.029	0.046	0.022	0.044	0.034
35	0.788	0.000	− 0.036	0.048	0.023	0.047	0.041
36	0.791	0.001	− 0.018	0.039	0.022	0.038	0.026
37	0.795	− 0.001	− 0.028	0.041	0.022	0.040	0.033
38	0.798	− 0.001	− 0.031	0.041	0.021	0.040	0.035
39	0.798	0.001	− 0.027	0.040	0.022	0.038	0.033
40	0.803	0.000	− 0.033	0.040	0.022	0.039	0.038
Average	0.745	0.000	0.001	0.048	0.023	0.047	0.028

#### Sensitivity analyses

**Fig. 4:**
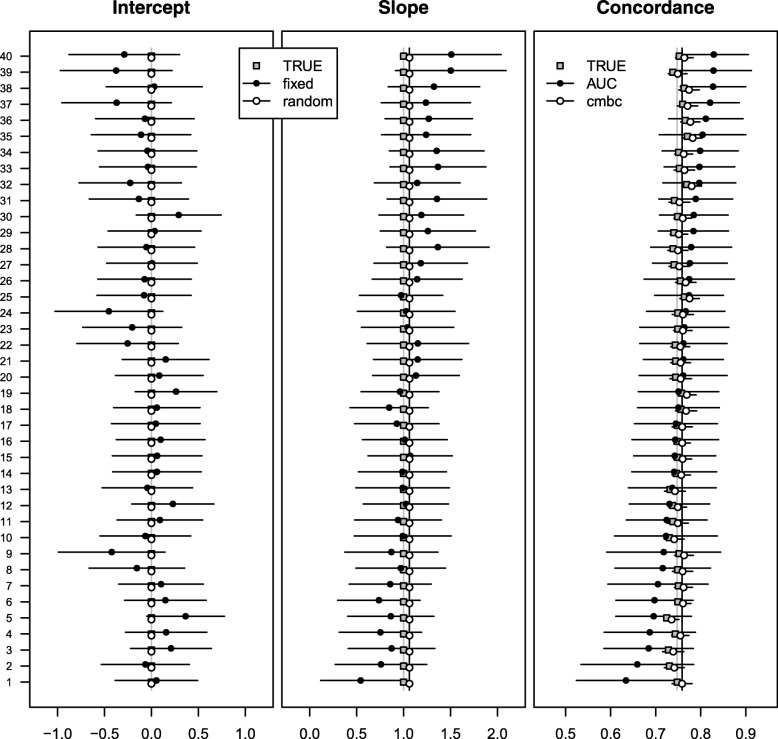
Performance measures across 40 centers of simulated 200 patients without between center heterogeneity in intercept and slope. Gray squares represent true values of intercept, slope, and concordance probability. Closed dots represent fixed effect intercept estimates; fixed effect slope estimates; and c-indexes in the first, second, and third panel, respectively. Open dots represent random effects intercept estimates; random effects slope estimates; and calibrated model-based concordance estimates in the first, second, and third panel, respectively. Gray vertical lines represent effect estimates of intercept and slope in the original regression model, together with the expected pooled concordance. Black vertical lines represent fixed effect estimates of intercept and slope in a multilevel regression model, together with the expected pooled concordance

We varied simulation settings to visualize the impact on our proposed approach. Without between-cluster heterogeneity of the true intercept and slope, the random effects estimates and the *c-mbc* were much closer to the true value than the fixed effect estimates and the c-index (Fig. 4). As a consequence of the unbiasedness of the *c-mbc*, the rmse of *c-mbc* was substantially lower compared to the c-index (Additional file 1: Table S3). When we doubled the number of patients in each cluster to 400, the standard deviation of the c-index, the bias of the *c-mbc*, and the average difference between the rmse of the *c-mbc* and the rmse of the c-index all were lower than in the simulations with 200 patients in each cluster (Additional file 1: Table S4).

We studied the impact on the simulation results when the regression model was misspecified and when the assumption of normally distributed calibration slopes was violated, respectively. To mimic model misspecification, we first generated binary outcomes based on a dichotomized version of the continuous baseline linear predictor *z*_*ik*_, i.e., from a Bernoulli distribution with success probability ${\left[1+\exp \left\{-\left(-0.5+{\gamma}_{0k}+2.75\ {\gamma}_{1k}\ {I}_{\left\{{z}_{ik}>1\right\}}\right)\right\}\right]}^{-1}$. The average intercept (− 0.5) and the average slope (2.75) of the outcome generation model were chosen such that the average intercept and slope estimated by the misspecified model based on continuous predictors *z*_*ik*_ were similar to the base-case scenario (− 2 and 1, respectively). Regardless of misspecification of the regression model, the rmse of the *c-mbc* was consistently lower than the rmse of the c-index (Additional file 1: Table S5). Second, we decreased the normally distributed calibration slopes in half of the clusters with 0.2 (weaker association between predictor and outcome) and increased the calibration slopes with 0.2 in the other half of the clusters (weaker association). Although the bias of the c-mbc was recognizable—upwards in the cluster with decreased calibration slope and downwards in the other half—the rmse of the *c-mbc* was again consistently lower than the rmse of the c-index (Additional file 1: Table S6).

**Fig. 5:**
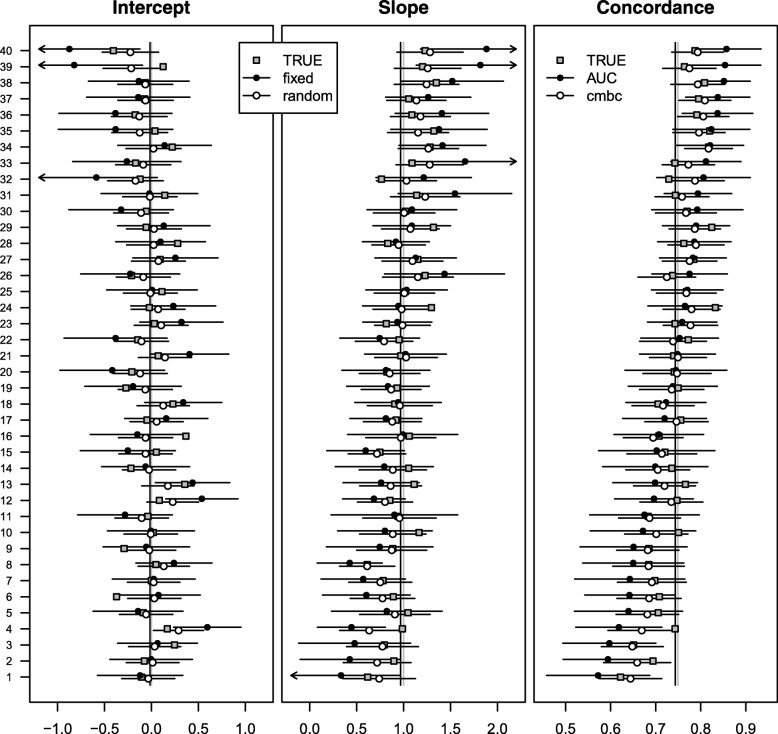
Performance measures across 40 centers of 200 simulated patients with varying case-mix heterogeneity. Gray squares represent true values of intercept, slope, and concordance probability. Closed dots represent fixed effect intercept estimates; fixed effect slope estimates; and c-indexes in the first, second, and third panel, respectively. Open dots represent random effects intercept estimates; random effects slope estimates; and calibrated model-based concordance estimates in the first, second, and third panel, respectively. Gray vertical lines represent effect estimates of intercept and slope in the original regression model, together with the expected pooled concordance. Black vertical lines represent fixed effect estimates of intercept and slope in a multilevel regression model, together with the expected pooled concordance

**Fig. 6:**
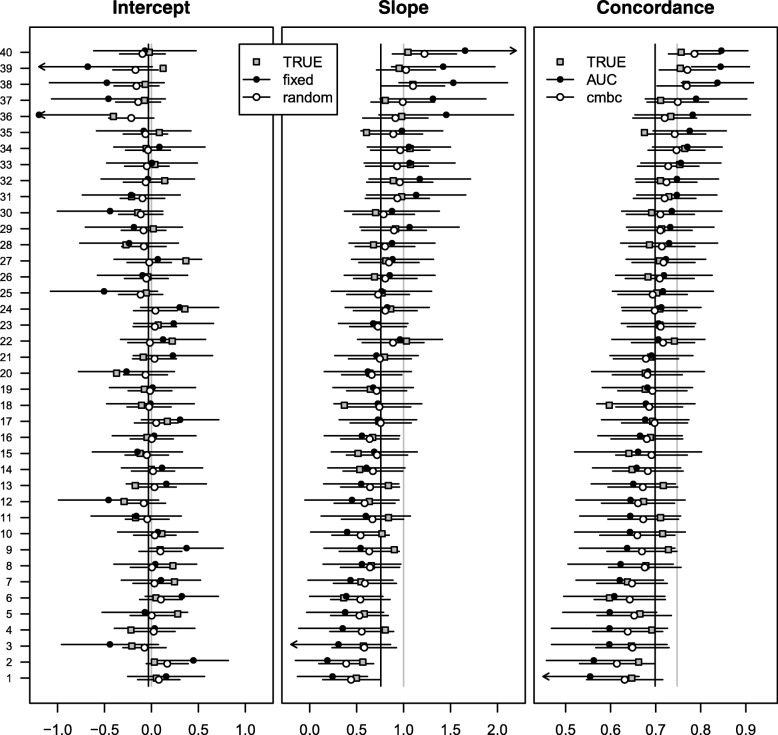
Performance measures across 40 centers of 200 simulated patients with reduced overall predictive ability. Gray squares represent true values of intercept, slope, and concordance probability. Closed dots represent fixed effect intercept estimates; fixed effect slope estimates; and c-indexes in the first, second, and third panel, respectively. Open dots represent random effects intercept estimates; random effects slope estimates; and calibrated model-based concordance estimates in the first, second, and third panel, respectively. Gray vertical lines represent effect estimates of intercept and slope in the original regression model, together with the expected pooled concordance. Black vertical lines represent fixed effect estimates of intercept and slope in a multilevel regression model, together with the expected pooled concordance

Finally, we varied the case-mix heterogeneity across clusters by drawing the standard deviation of the normally distributed predictor in cluster *k* (*z*_*ik*_) from a uniform distribution between 0.75 and 1.25, and we reduced overall predictive ability by a true slope of 0.75. Both scenarios were well presented in cluster-specific estimates, by more variation in *c-mbc* (Fig. 5) and lower mean *c-mbc* (Fig. 6), respectively.

## Discussion

We proposed a new approach for assessing discriminative ability of prediction models in clustered data. The measure is a modification of the previously proposed calibrated model-based concordance (*c-mbc*) that is the expected concordance under the assumption that the estimates of calibration intercept and slope of the prediction model are true. The *c-mbc* for clustered data uses the random effect estimates of the calibration intercept and slope per cluster provided by a multilevel logistic regression model with the linear predictor as only covariate. The *c-mbc* was less extreme in distribution than the c-index in a case study. In simulations with a heterogeneous calibration slope, the random effect estimates of calibration intercept and slope and thus the *c-mbc* were biased, but less variable than the unbiased fixed effect estimates and the c-index. The trade-off between bias and variance resulted in a generally lower root mean squared error of the *c-mbc* compared to the c-index.

We compared the *c-mbc* based on random effect estimates of the calibration intercept and slope with the c-index. The comparison is basically between a random effect concordance probability estimator and a fixed effect concordance probability estimator, because the c-index is asymptotically equivalent to the *c-mbc* based on fixed effect estimates of the calibration intercept and slope [6]. This explains the observed variance bias trade-off which is typical for the choice between fixed effect and random effect estimates. It is well recognized that unbiasedness is not the only property of an estimator that is important and that much could be gained by compromising unbiasedness to improve the precision of an estimator [10, 22].

We and others have recently suggested summarizing the discriminative ability with random-effects meta-analysis of the cluster-specific c-index, because the discriminative ability often varies between clusters of patients [8, 23, 24]. Random effects meta-analytic techniques inform about the mean and the variation in cluster-specific concordance probabilities, ideally with a prediction interval [25]. However, meta-analytic techniques do not add information about the concordance probability in individual clusters. The techniques proposed in this paper enhance the assessment of discriminative ability in individual clusters of patients.

The patients in our case study were clustered in hospitals. A comparable type of clustering may occur in patients treated in different countries or in patients treated by different caregivers in the same center. Similarly, in public health research, the study population is often clustered in geographical regions like countries, municipalities, or neighborhoods. Patients in an individual patient data (IPD) meta-analysis are also clustered in studies. In general, we recommend to always exploit the non-randomly clustered nature of a dataset, through analysis and reporting of the variation in prediction model performance across clusters. Hence, we obtain more insight into the generalizability of a prediction model across different settings. Even at internal validation, the variation in model performance across non-random clusters is more informative than the quantification of a model’s internal validity based on random sampling techniques.

We focused on measuring the performance of logistic regression models in clustered data, using multilevel logistic regression and the calibration intercept, the calibration slope, the c-index, and the *c-mbc*. This methodology could easily be extended to proportional hazards regression models, based on mixed effects Cox models or shared frailty models, and similar definitions of the calibration slope, the c-index, and the *c-mbc* in survival data [4, 6, 26].

We initially simulated validation studies of a logistic regression with moderate heterogeneity in true intercepts and slopes across 40 rather small clusters of 200 patients. Obviously, the difference in the rmse of the *c-mbc* compared to the c-index depends on the characteristics of the setting. With negligible heterogeneity in true intercepts and slopes, the difference in rsme was higher. With growing numbers of patients per cluster, the difference in rsme was lower. Ultimately, the *c-mbc* converges to the c-index with increasing numbers of patients per cluster, because the random effect estimates converge to the fixed effect estimates [6].

The proposed approach depends on the ability of a multilevel regression model to estimate the between-cluster variances of the intercept and the slope. The minimum number of clusters needed to estimate these variances is in the order of 10 but depends on the specific setting [9].

## Conclusions

The *c-mbc*, based on random effect estimates of the calibration intercept and slope, resulted in a generally lower root mean squared error compared to the c-index. The *c-mbc* is an attractive alternative to the c-index as measure of discriminative ability in clustered data when clusters are of limited size.

### Additional file

Additional file 1: **Table S1.** Cluster sizes and performance measures across 19 European centers of the CRASH trial. **Table S2.** Cluster sizes and performance measures at external validation across 36 non-European centers of the CRASH trial. **Table S3.** Simulation characteristics (2000 replications) of c-index and *c-mbc* across 40 centers of 200 patients without between center heterogeneity in intercept and slope. **Table S4.** Simulation characteristics (2000 replications) of c-index and *c-mbc* across 40 centers of 400 patients. **Table S5.** Simulation characteristics (2000 replications) of c-index and c-mbc across 40 centers of 200 patients when the regression model was misspecified. **Table S6.** Simulation characteristics (2000 replications) of c-index and *c-mbc* across 40 centers of 200 patients when the calibration slopes were not normally distributed. (DOCX 92 kb)

## Appendix

The model-based concordance (*mbc*) is a model-based estimator of the concordance probability [6]. The concordance probability is defined as the probability that a model predicts for two randomly chosen patients with different outcomes and a higher risk for the patient with poorer outcome. For a given patient population (or cluster of patients), it is the probability that a randomly selected patient pair has concordant predictions and outcomes, divided by the probability that their outcomes are different (not “tied”). Patient *i* has binary outcome *Y*_*i*_, baseline characteristics vector *x*_*i*_, linear predictor ${x}_i^T\beta$ of a logistic regression model, and prediction ${p}_i={\mathrm{logit}}^{-1}\left({\beta}_0+{x}_i^T\beta \right)$. The probability that a randomly selected patient pair has concordant predictions and outcomes is [27]

3 $$ P\left(\mathrm{concordant}\right)=\frac{1}{n\left(n-1\right)}{\sum}_i{\sum}_{j\ne i}\left[I\left({p}_i<{p}_j\right)P\left({Y}_i<{Y}_j\right)+I\left({p}_i>{p}_j\right)P\left({Y}_i>{Y}_j\right)\right] $$

Similarly, the probability that a randomly selected patient pair has unequal outcomes is

4 $$ P\left(\mathrm{unequal}\ Y\right)=\frac{1}{n\left(n-1\right)}\sum \limits_i\sum \limits_{j\ne i}\left[P\left({Y}_i<{Y}_j\right)+P\left({Y}_i>{Y}_j\right)\right] $$

Thus, the concordance probability CP in a patient population is obtained by dividing the probabilities of Eqs. 3 and 4:

5 $$ \mathrm{CP}=\frac{\sum \limits_i\sum \limits_{j\ne i}\left[I\left({p}_i<{p}_j\right)P\left({Y}_i<{Y}_j\right)+I\left({p}_i>{p}_j\right)P\left({Y}_i>{Y}_j\right)\right]}{\sum \limits_i\sum \limits_{j\ne i}\left[P\left({Y}_i<{Y}_j\right)+P\left({Y}_i>{Y}_j\right)\right]} $$

For a logistic regression model, the model-based probabilities *P*(*Y*_*i*_ < *Y*_*j*_) are

6 $$ P\left({Y}_i<{Y}_j\right)=P\left({Y}_i=0\right)P\left({Y}_j=1\right)=\frac{1}{1+{e}^{\beta_0+{x}_i^T\beta }}\ \frac{1}{1+{e}^{-\left({\beta}_0+{x}_j^T\beta \right)}} $$

Combining Eqs. 5 and 6 and replacing *I*(*p*_*i*_ < *p*_*j*_) by $I\left({x}_i^T\beta <{x}_j^T\beta \right)$ because the predictions are an increasing function of the linear predictor result in the model-based concordance (*mbc*) for logistic regression models:

7 $$ mbc\left({\beta}_0+ X\beta \right)=\frac{\sum_i{\sum}_{j\ne i}\left[\frac{I\left({x}_i^T\beta <{x}_j^T\beta \right)}{\left(1+{e}^{\beta_0+{x}_i^T\beta}\right)\left(1+{e}^{-\left({\beta}_0+{x}_j^T\beta \right)}\right)}+\frac{I\left({x}_i^T\beta >{x}_j^T\beta \right)}{\left(1+{e}^{-\left({\beta}_0+{x}_i^T\beta \right)}\right)\left(1+{e}^{\beta_0+{x}_j^T\beta}\right)}\right]}{\sum_i{\sum}_{j\ne i}\left[\frac{1}{\left(1+{e}^{\beta_0+{x}_i^T\beta}\right)\left(1+{e}^{-\left({\beta}_0+{x}_j^T\beta \right)}\right)}+\frac{1}{\left(1+{e}^{-\left({\beta}_0+{x}_i^T\beta \right)}\right)\left(1+{e}^{\beta_0+{x}_j^T\beta}\right)}\right]} $$

When model predictions may be equal for some combinations of *i* and *j*, e.g., when *x* is a binary marker, we can generalize 5 by using *I*(*p*_*i*_ ≤ *p*_*j*_) instead of *I*(*p*_*i*_ < *p*_*j*_).
