# Supplementary material for: The calibrated model-based concordance improved assessment of discriminative ability in patient clusters of limited sample size
Source: Diagn Progn Res. 2019 Jun 6;3:11. doi: 10.1186/s41512-019-0055-8 (PMC6551913; doi:10.1186/s41512-019-0055-8)
Supplement: Supplementary file 1 — Table S1. Cluster sizes and performance measures across 19 European centers of the CRASH trial. Table S2. Cluster sizes and performance measures at external validation across 36 non-European centers of the CRASH trial. Table S3. Simulation characteristics (2000 replications) of c-index and c-mbc across 40 centers of 200 patients without between center heterogeneity in intercept and slope. Table S4. Simulation characteristics (2000 replications) of c-index and c-mbc across 40 centers of 400 patients. Table S5. Simulation characteristics (2000 replications) of c-index and c-mbc across 40 centers of 200 patients when the regression model was misspecified. Table S6. Simulation characteristics (2000 replications) of c-index and c-mbc across 40 centers of 200 patients when the calibration slopes were not normally distributed. (DOCX 92 kb) [file 41512_2019_55_MOESM1_ESM.docx]

Additional file 1: Table S1 Cluster sizes and p**erformance measures across 19 European centers of the CRASH trial.** Fixed effect intercept estimates, fixed effect slope estimates and c-indexes are in the fourth, sixth and eighth column respectively. Random effects intercept estimates, random effects slope estimates and calibrated model-based concordance estimates are in the fifth, seventh and ninth column respectively.

| Cluster | Patients | Events | Intercept | |  | Slope | |  | Concordance | |
| --- | --- | --- | --- | --- | --- | --- | --- | --- | --- | --- |
|  |  |  | fixed | random |  | fixed | random |  | c-index | c-mbc |
| 1 | 103 | 21 | 1.9 (0.70) | 0.6 (0.50) |  | 0.33 (0.21) | 0.75 (0.13) |  | 0.63 (0.07) | 0.73 (0.04) |
| 2 | 71 | 14 | 1.2 (0.76) | 0.4 (0.53) |  | 0.57 (0.23) | 0.85 (0.14) |  | 0.69 (0.09) | 0.78 (0.04) |
| 3 | 122 | 31 | 0.7 (0.65) | 0.3 (0.48) |  | 0.75 (0.18) | 0.87 (0.12) |  | 0.76 (0.05) | 0.80 (0.03) |
| 4 | 31 | 19 | 2.4 (1.00) | 1.4 (0.61) |  | 0.69 (0.29) | 0.87 (0.14) |  | 0.77 (0.09) | 0.83 (0.04) |
| 5 | 66 | 25 | 1.2 (0.75) | 0.7 (0.54) |  | 0.73 (0.20) | 0.87 (0.13) |  | 0.79 (0.06) | 0.83 (0.03) |
| 6 | 57 | 25 | 1.1 (0.87) | 0.6 (0.57) |  | 0.77 (0.22) | 0.90 (0.13) |  | 0.80 (0.06) | 0.83 (0.03) |
| 7 | 34 | 12 | -0.7 (1.82) | 0.1 (0.66) |  | 1.17 (0.47) | 0.99 (0.15) |  | 0.81 (0.07) | 0.80 (0.04) |
| 8 | 41 | 13 | 0.2 (1.23) | 0.4 (0.59) |  | 1.08 (0.37) | 0.97 (0.15) |  | 0.82 (0.06) | 0.81 (0.04) |
| 9 | 23 | 11 | -0.2 (1.99) | 0.3 (0.69) |  | 1.15 (0.51) | 0.98 (0.15) |  | 0.82 (0.08) | 0.82 (0.04) |
| 10 | 110 | 25 | -0.1 (0.79) | -0.1 (0.52) |  | 0.91 (0.21) | 0.95 (0.13) |  | 0.82 (0.04) | 0.83 (0.03) |
| 11 | 30 | 11 | 0.1 (1.31) | 0.5 (0.62) |  | 1.21 (0.43) | 0.97 (0.14) |  | 0.84 (0.09) | 0.86 (0.04) |
| 12 | 61 | 13 | -0.8 (1.17) | -0.3 (0.60) |  | 1.09 (0.29) | 1.00 (0.14) |  | 0.84 (0.07) | 0.85 (0.03) |
| 13 | 45 | 14 | 0.3 (1.14) | 0.3 (0.61) |  | 0.93 (0.30) | 0.95 (0.14) |  | 0.85 (0.06) | 0.84 (0.04) |
| 14 | 50 | 16 | -0.4 (1.28) | 0.2 (0.60) |  | 1.16 (0.35) | 1.00 (0.14) |  | 0.86 (0.05) | 0.84 (0.03) |
| 15 | 53 | 18 | -0.2 (1.11) | 0.1 (0.60) |  | 1.05 (0.28) | 0.98 (0.14) |  | 0.87 (0.05) | 0.86 (0.03) |
| 16 | 181 | 107 | 1.1 (0.49) | 1.4 (0.36) |  | 1.14 (0.17) | 0.99 (0.11) |  | 0.88 (0.02) | 0.87 (0.02) |
| 17 | 116 | 16 | -1.3 (1.02) | -0.3 (0.49) |  | 1.41 (0.32) | 1.07 (0.13) |  | 0.89 (0.03) | 0.81 (0.03) |
| 18 | 28 | 13 | -0.5 (1.67) | 0.1 (0.68) |  | 1.14 (0.40) | 0.99 (0.15) |  | 0.90 (0.06) | 0.88 (0.03) |
| 19 | 494 | 41 | -2.1 (0.61) | -1.5 (0.41) |  | 1.33 (0.17) | 1.17 (0.11) |  | 0.92 (0.02) | 0.86 (0.02) |

Table S2 Cluster sizes and p**erformance measures at external validation across 36 non-European centers of the CRASH trial.** Fixed effect intercept estimates, fixed effect slope estimates and c-indexes are in the fourth, sixth and eighth column respectively. Random effects intercept estimates, random effects slope estimates and calibrated model-based concordance estimates are in the fifth, seventh and ninth column respectively.

| Cluster | Patients | Events | Intercept | |  | Slope | |  | Concordance | |
| --- | --- | --- | --- | --- | --- | --- | --- | --- | --- | --- |
|  |  |  | fixed | random |  | fixed | random |  | c-index | c-mbc |
| 1 | 27 | 14 | 3.1 (0.85) | 2.4 (0.49) |  | 0.61 (0.39) | 0.78 (0.12) |  | 0.64 (0.11) | 0.73 (0.04) |
| 2 | 29 | 15 | 2.5 (0.95) | 2.0 (0.52) |  | 0.73 (0.37) | 0.84 (0.13) |  | 0.66 (0.10) | 0.76 (0.04) |
| 3 | 32 | 19 | 3.6 (0.73) | 2.6 (0.48) |  | 0.40 (0.28) | 0.74 (0.12) |  | 0.66 (0.10) | 0.75 (0.04) |
| 4 | 276 | 84 | 2.2 (0.31) | 1.7 (0.27) |  | 0.50 (0.11) | 0.70 (0.09) |  | 0.67 (0.03) | 0.71 (0.02) |
| 5 | 25 | 14 | 2.2 (1.23) | 1.8 (0.57) |  | 0.78 (0.42) | 0.87 (0.13) |  | 0.69 (0.10) | 0.77 (0.04) |
| 6 | 71 | 30 | 2.2 (0.59) | 1.8 (0.41) |  | 0.67 (0.21) | 0.83 (0.11) |  | 0.71 (0.07) | 0.77 (0.03) |
| 7 | 131 | 63 | 2.5 (0.42) | 2.1 (0.33) |  | 0.65 (0.15) | 0.77 (0.10) |  | 0.73 (0.05) | 0.76 (0.03) |
| 8 | 96 | 61 | 2.9 (0.53) | 2.6 (0.37) |  | 0.71 (0.20) | 0.77 (0.11) |  | 0.74 (0.05) | 0.76 (0.03) |
| 9 | 66 | 12 | 1.2 (0.80) | 0.6 (0.50) |  | 0.57 (0.25) | 0.90 (0.12) |  | 0.74 (0.07) | 0.79 (0.04) |
| 10 | 133 | 50 | 2.0 (0.43) | 1.7 (0.33) |  | 0.74 (0.16) | 0.83 (0.10) |  | 0.75 (0.05) | 0.77 (0.03) |
| 11 | 46 | 15 | 1.1 (1.02) | 1.2 (0.50) |  | 0.93 (0.35) | 0.93 (0.13) |  | 0.75 (0.08) | 0.76 (0.03) |
| 12 | 466 | 146 | 1.5 (0.26) | 1.5 (0.21) |  | 0.90 (0.11) | 0.90 (0.08) |  | 0.75 (0.02) | 0.75 (0.02) |
| 13 | 48 | 24 | 1.9 (0.90) | 1.8 (0.47) |  | 0.88 (0.33) | 0.87 (0.12) |  | 0.75 (0.07) | 0.75 (0.04) |
| 14 | 131 | 33 | 1.0 (0.58) | 1.2 (0.34) |  | 1.05 (0.25) | 0.95 (0.11) |  | 0.76 (0.05) | 0.74 (0.03) |
| 15 | 47 | 24 | 2.1 (0.77) | 1.9 (0.47) |  | 0.84 (0.28) | 0.86 (0.12) |  | 0.77 (0.07) | 0.79 (0.03) |
| 16 | 50 | 17 | 0.9 (0.94) | 1.7 (0.42) |  | 1.45 (0.49) | 0.90 (0.12) |  | 0.77 (0.08) | 0.73 (0.04) |
| 17 | 94 | 28 | 1.0 (0.63) | 0.9 (0.43) |  | 0.85 (0.19) | 0.92 (0.11) |  | 0.78 (0.06) | 0.82 (0.03) |
| 18 | 78 | 19 | 0.2 (0.86) | 0.5 (0.48) |  | 0.99 (0.26) | 0.98 (0.12) |  | 0.79 (0.06) | 0.80 (0.03) |
| 19 | 125 | 49 | 1.1 (0.61) | 1.1 (0.40) |  | 0.90 (0.19) | 0.92 (0.11) |  | 0.79 (0.04) | 0.78 (0.03) |
| 20 | 124 | 36 | 0.4 (0.67) | 1.6 (0.31) |  | 1.62 (0.35) | 0.96 (0.11) |  | 0.79 (0.05) | 0.70 (0.02) |
| 21 | 60 | 30 | 1.2 (0.91) | 1.5 (0.48) |  | 1.04 (0.30) | 0.92 (0.12) |  | 0.80 (0.06) | 0.78 (0.03) |
| 22 | 107 | 43 | 1.4 (0.56) | 1.4 (0.39) |  | 0.89 (0.19) | 0.90 (0.11) |  | 0.80 (0.04) | 0.80 (0.03) |
| 23 | 32 | 20 | 1.8 (1.09) | 1.7 (0.57) |  | 0.91 (0.35) | 0.88 (0.13) |  | 0.80 (0.08) | 0.84 (0.04) |
| 24 | 52 | 17 | 0.5 (1.03) | 1.4 (0.45) |  | 1.38 (0.43) | 0.95 (0.12) |  | 0.80 (0.07) | 0.75 (0.03) |
| 25 | 37 | 16 | 1.1 (1.07) | 1.6 (0.50) |  | 1.14 (0.42) | 0.91 (0.13) |  | 0.80 (0.07) | 0.76 (0.04) |
| 26 | 65 | 38 | 2.2 (0.66) | 2.2 (0.42) |  | 0.90 (0.25) | 0.84 (0.11) |  | 0.80 (0.05) | 0.80 (0.03) |
| 27 | 96 | 20 | 0.2 (0.76) | 0.8 (0.40) |  | 1.24 (0.31) | 1.00 (0.12) |  | 0.81 (0.06) | 0.76 (0.03) |
| 28 | 27 | 13 | 0.5 (1.43) | 1.3 (0.58) |  | 1.22 (0.46) | 0.94 (0.13) |  | 0.81 (0.09) | 0.81 (0.04) |
| 29 | 44 | 20 | 1.0 (0.96) | 1.6 (0.49) |  | 1.17 (0.35) | 0.92 (0.12) |  | 0.83 (0.06) | 0.79 (0.03) |
| 30 | 34 | 15 | 0.7 (1.33) | 2.0 (0.46) |  | 1.88 (0.85) | 0.87 (0.12) |  | 0.84 (0.07) | 0.75 (0.05) |
| 31 | 71 | 20 | 1.4 (0.58) | 1.4 (0.41) |  | 0.84 (0.23) | 0.90 (0.11) |  | 0.84 (0.05) | 0.80 (0.04) |
| 32 | 56 | 26 | 0.9 (0.87) | 1.5 (0.46) |  | 1.18 (0.32) | 0.94 (0.12) |  | 0.84 (0.05) | 0.82 (0.03) |
| 33 | 34 | 13 | 1.1 (1.00) | 1.3 (0.53) |  | 0.98 (0.35) | 0.92 (0.13) |  | 0.84 (0.07) | 0.83 (0.04) |
| 34 | 44 | 12 | -1.4 (1.62) | 1.1 (0.48) |  | 2.02 (0.65) | 0.98 (0.13) |  | 0.84 (0.06) | 0.72 (0.03) |
| 35 | 185 | 19 | -1.9 (0.90) | -0.7 (0.45) |  | 1.38 (0.25) | 1.13 (0.12) |  | 0.90 (0.04) | 0.84 (0.03) |
| 36 | 96 | 19 | -2.0 (1.20) | -0.1 (0.48) |  | 1.65 (0.36) | 1.11 (0.12) |  | 0.94 (0.03) | 0.87 (0.03) |

Table S3 Simulation characteristics (2,000 replications) of c-index and c-mbc across 40 centers of 200 patients **without between center heterogeneity in intercept and slope.**

|  | True | Bias | | SD | | rmse | |
| --- | --- | --- | --- | --- | --- | --- | --- |
| Cluster | concordance | c-index | c-mbc | c-index | c-mbc | c-index | c-mbc |
| 1 | 0.746 | 0.001 | 0.001 | 0.047 | 0.014 | 0.046 | 0.009 |
| 2 | 0.746 | -0.002 | 0.000 | 0.049 | 0.014 | 0.048 | 0.009 |
| 3 | 0.746 | 0.001 | 0.001 | 0.049 | 0.014 | 0.048 | 0.009 |
| 4 | 0.746 | 0.000 | 0.000 | 0.046 | 0.014 | 0.045 | 0.009 |
| 5 | 0.746 | 0.002 | 0.001 | 0.048 | 0.014 | 0.047 | 0.009 |
| 6 | 0.746 | 0.000 | 0.000 | 0.048 | 0.014 | 0.047 | 0.009 |
| 7 | 0.746 | 0.000 | 0.000 | 0.049 | 0.014 | 0.047 | 0.009 |
| 8 | 0.746 | 0.002 | 0.001 | 0.049 | 0.014 | 0.047 | 0.009 |
| 9 | 0.746 | -0.001 | 0.000 | 0.049 | 0.014 | 0.047 | 0.009 |
| 10 | 0.746 | 0.001 | 0.000 | 0.048 | 0.014 | 0.047 | 0.009 |
| 11 | 0.746 | -0.001 | 0.000 | 0.047 | 0.014 | 0.046 | 0.009 |
| 12 | 0.746 | 0.001 | 0.000 | 0.049 | 0.014 | 0.047 | 0.009 |
| 13 | 0.746 | 0.001 | 0.001 | 0.047 | 0.014 | 0.046 | 0.009 |
| 14 | 0.746 | 0.001 | 0.000 | 0.048 | 0.014 | 0.046 | 0.009 |
| 15 | 0.746 | 0.000 | 0.000 | 0.048 | 0.014 | 0.047 | 0.009 |
| 16 | 0.746 | -0.002 | 0.000 | 0.046 | 0.013 | 0.045 | 0.009 |
| 17 | 0.746 | 0.002 | 0.001 | 0.046 | 0.014 | 0.045 | 0.009 |
| 18 | 0.746 | 0.000 | 0.000 | 0.048 | 0.014 | 0.047 | 0.009 |
| 19 | 0.746 | 0.001 | 0.000 | 0.049 | 0.014 | 0.047 | 0.009 |
| 20 | 0.746 | 0.000 | 0.000 | 0.047 | 0.014 | 0.046 | 0.009 |
| 21 | 0.746 | 0.002 | 0.000 | 0.048 | 0.014 | 0.047 | 0.009 |
| 22 | 0.746 | 0.001 | 0.000 | 0.047 | 0.014 | 0.046 | 0.009 |
| 23 | 0.746 | 0.000 | 0.001 | 0.047 | 0.014 | 0.046 | 0.009 |
| 24 | 0.746 | 0.001 | 0.000 | 0.047 | 0.013 | 0.046 | 0.009 |
| 25 | 0.746 | 0.001 | 0.001 | 0.048 | 0.014 | 0.046 | 0.009 |
| 26 | 0.746 | 0.000 | 0.000 | 0.049 | 0.014 | 0.048 | 0.009 |
| 27 | 0.746 | 0.000 | 0.000 | 0.048 | 0.014 | 0.047 | 0.009 |
| 28 | 0.746 | -0.002 | 0.000 | 0.047 | 0.013 | 0.046 | 0.009 |
| 29 | 0.746 | 0.000 | 0.001 | 0.048 | 0.014 | 0.047 | 0.009 |
| 30 | 0.747 | 0.000 | 0.000 | 0.048 | 0.014 | 0.047 | 0.009 |
| 31 | 0.747 | 0.002 | 0.001 | 0.048 | 0.014 | 0.047 | 0.009 |
| 32 | 0.747 | 0.001 | 0.000 | 0.047 | 0.014 | 0.046 | 0.009 |
| 33 | 0.747 | 0.001 | 0.000 | 0.048 | 0.014 | 0.046 | 0.009 |
| 34 | 0.747 | 0.002 | 0.001 | 0.047 | 0.014 | 0.046 | 0.009 |
| 35 | 0.747 | -0.001 | 0.000 | 0.048 | 0.014 | 0.047 | 0.009 |
| 36 | 0.747 | 0.000 | 0.000 | 0.048 | 0.014 | 0.047 | 0.009 |
| 37 | 0.747 | 0.000 | 0.000 | 0.048 | 0.014 | 0.046 | 0.009 |
| 38 | 0.747 | 0.001 | 0.001 | 0.047 | 0.014 | 0.046 | 0.009 |
| 39 | 0.747 | -0.002 | 0.000 | 0.047 | 0.014 | 0.046 | 0.009 |
| 40 | 0.747 | 0.001 | 0.001 | 0.047 | 0.014 | 0.046 | 0.009 |
|  |  |  |  |  |  |  |  |
| Average | 0.746 | 0.000 | 0.000 | 0.048 | 0.014 | 0.047 | 0.009 |

Table S4 Simulation characteristics (2,000 replications) of c-index and c-mbc across 40 centers of 400 patients.

|  | True | Bias | | SD | | rmse | |
| --- | --- | --- | --- | --- | --- | --- | --- |
| Cluster | concordance | c-index | c-mbc | c-index | c-mbc | c-index | c-mbc |
| 1 | 0.664 | 0.000 | 0.038 | 0.039 | 0.025 | 0.039 | 0.045 |
| 2 | 0.666 | -0.001 | 0.033 | 0.043 | 0.025 | 0.042 | 0.041 |
| 3 | 0.695 | 0.000 | 0.024 | 0.038 | 0.022 | 0.038 | 0.032 |
| 4 | 0.699 | -0.001 | 0.017 | 0.039 | 0.022 | 0.039 | 0.027 |
| 5 | 0.703 | 0.000 | 0.019 | 0.037 | 0.021 | 0.037 | 0.028 |
| 6 | 0.705 | 0.001 | 0.025 | 0.035 | 0.022 | 0.034 | 0.032 |
| 7 | 0.710 | -0.001 | 0.016 | 0.037 | 0.021 | 0.036 | 0.025 |
| 8 | 0.712 | -0.001 | 0.021 | 0.033 | 0.021 | 0.033 | 0.029 |
| 9 | 0.712 | 0.000 | 0.009 | 0.038 | 0.021 | 0.038 | 0.022 |
| 10 | 0.718 | 0.001 | 0.015 | 0.034 | 0.020 | 0.034 | 0.024 |
| 11 | 0.726 | -0.001 | 0.000 | 0.039 | 0.020 | 0.038 | 0.019 |
| 12 | 0.727 | 0.000 | 0.006 | 0.036 | 0.020 | 0.035 | 0.020 |
| 13 | 0.727 | 0.001 | -0.003 | 0.039 | 0.021 | 0.038 | 0.020 |
| 14 | 0.727 | 0.001 | 0.015 | 0.033 | 0.020 | 0.032 | 0.024 |
| 15 | 0.732 | 0.002 | 0.006 | 0.035 | 0.019 | 0.034 | 0.019 |
| 16 | 0.735 | 0.001 | -0.003 | 0.038 | 0.020 | 0.038 | 0.019 |
| 17 | 0.735 | 0.000 | 0.004 | 0.035 | 0.019 | 0.035 | 0.018 |
| 18 | 0.738 | 0.000 | -0.001 | 0.037 | 0.020 | 0.036 | 0.018 |
| 19 | 0.741 | 0.000 | 0.005 | 0.032 | 0.018 | 0.032 | 0.018 |
| 20 | 0.743 | 0.000 | 0.006 | 0.033 | 0.019 | 0.032 | 0.018 |
| 21 | 0.750 | 0.000 | 0.002 | 0.032 | 0.018 | 0.031 | 0.017 |
| 22 | 0.751 | 0.001 | -0.003 | 0.033 | 0.019 | 0.033 | 0.017 |
| 23 | 0.755 | 0.001 | 0.007 | 0.029 | 0.019 | 0.028 | 0.019 |
| 24 | 0.755 | 0.000 | -0.006 | 0.033 | 0.019 | 0.032 | 0.018 |
| 25 | 0.757 | -0.002 | -0.007 | 0.034 | 0.018 | 0.033 | 0.018 |
| 26 | 0.757 | 0.000 | -0.012 | 0.034 | 0.018 | 0.034 | 0.021 |
| 27 | 0.763 | 0.000 | -0.013 | 0.034 | 0.019 | 0.033 | 0.021 |
| 28 | 0.764 | 0.000 | -0.013 | 0.034 | 0.018 | 0.033 | 0.021 |
| 29 | 0.764 | 0.000 | 0.002 | 0.029 | 0.019 | 0.028 | 0.017 |
| 30 | 0.770 | 0.000 | -0.005 | 0.030 | 0.018 | 0.029 | 0.017 |
| 31 | 0.772 | 0.000 | -0.008 | 0.030 | 0.017 | 0.029 | 0.017 |
| 32 | 0.775 | 0.000 | -0.011 | 0.030 | 0.018 | 0.029 | 0.019 |
| 33 | 0.780 | 0.001 | -0.009 | 0.029 | 0.018 | 0.028 | 0.018 |
| 34 | 0.786 | 0.000 | -0.023 | 0.032 | 0.018 | 0.031 | 0.028 |
| 35 | 0.788 | 0.000 | -0.032 | 0.033 | 0.019 | 0.032 | 0.036 |
| 36 | 0.791 | 0.000 | -0.012 | 0.027 | 0.018 | 0.026 | 0.019 |
| 37 | 0.795 | 0.000 | -0.020 | 0.030 | 0.018 | 0.029 | 0.026 |
| 38 | 0.798 | 0.000 | -0.020 | 0.028 | 0.018 | 0.027 | 0.025 |
| 39 | 0.798 | 0.000 | -0.023 | 0.029 | 0.018 | 0.028 | 0.028 |
| 40 | 0.803 | 0.000 | -0.025 | 0.029 | 0.018 | 0.028 | 0.030 |
|  |  |  |  |  |  |  |  |
| Average | 0.745 | 0.000 | 0.001 | 0.034 | 0.019 | 0.033 | 0.023 |

Table S5 Simulation characteristics (2,000 replications) of c-index and c-mbc across 40 centers of 200 patients when the regression model was misspecified.

|  | True | Bias | | SD | | rmse | |
| --- | --- | --- | --- | --- | --- | --- | --- |
| Cluster | concordance | c-index | c-mbc | c-index | c-mbc | c-index | c-mbc |
| 1 | 0.653 | 0.001 | 0.044 | 0.069 | 0.039 | 0.068 | 0.059 |
| 2 | 0.657 | 0.001 | 0.039 | 0.074 | 0.039 | 0.073 | 0.056 |
| 3 | 0.689 | 0.002 | 0.029 | 0.062 | 0.035 | 0.060 | 0.045 |
| 4 | 0.696 | 0.002 | 0.030 | 0.060 | 0.036 | 0.056 | 0.046 |
| 5 | 0.697 | 0.001 | 0.021 | 0.067 | 0.036 | 0.065 | 0.042 |
| 6 | 0.700 | 0.003 | 0.024 | 0.063 | 0.035 | 0.061 | 0.042 |
| 7 | 0.703 | 0.001 | 0.028 | 0.057 | 0.036 | 0.056 | 0.045 |
| 8 | 0.707 | 0.001 | 0.021 | 0.063 | 0.035 | 0.061 | 0.041 |
| 9 | 0.714 | 0.001 | 0.019 | 0.061 | 0.035 | 0.059 | 0.040 |
| 10 | 0.715 | 0.000 | 0.011 | 0.068 | 0.035 | 0.066 | 0.037 |
| 11 | 0.721 | -0.001 | 0.020 | 0.057 | 0.035 | 0.054 | 0.039 |
| 12 | 0.729 | 0.000 | 0.009 | 0.062 | 0.033 | 0.060 | 0.034 |
| 13 | 0.733 | 0.001 | 0.001 | 0.067 | 0.034 | 0.065 | 0.034 |
| 14 | 0.734 | 0.001 | 0.009 | 0.060 | 0.033 | 0.058 | 0.034 |
| 15 | 0.736 | 0.003 | -0.002 | 0.067 | 0.033 | 0.066 | 0.034 |
| 16 | 0.738 | 0.001 | 0.007 | 0.061 | 0.034 | 0.058 | 0.033 |
| 17 | 0.740 | 0.001 | 0.013 | 0.054 | 0.033 | 0.052 | 0.034 |
| 18 | 0.741 | 0.000 | 0.009 | 0.058 | 0.033 | 0.055 | 0.033 |
| 19 | 0.742 | 0.001 | 0.017 | 0.051 | 0.033 | 0.047 | 0.036 |
| 20 | 0.743 | 0.000 | -0.002 | 0.062 | 0.032 | 0.060 | 0.032 |
| 21 | 0.744 | 0.002 | 0.003 | 0.060 | 0.032 | 0.058 | 0.032 |
| 22 | 0.749 | 0.001 | 0.009 | 0.053 | 0.031 | 0.050 | 0.031 |
| 23 | 0.751 | 0.001 | 0.014 | 0.051 | 0.033 | 0.048 | 0.035 |
| 24 | 0.756 | 0.001 | 0.001 | 0.057 | 0.031 | 0.055 | 0.031 |
| 25 | 0.762 | 0.001 | -0.002 | 0.056 | 0.031 | 0.054 | 0.031 |
| 26 | 0.764 | 0.002 | -0.002 | 0.057 | 0.032 | 0.054 | 0.031 |
| 27 | 0.768 | 0.001 | 0.003 | 0.051 | 0.031 | 0.048 | 0.030 |
| 28 | 0.769 | 0.003 | -0.008 | 0.058 | 0.031 | 0.055 | 0.031 |
| 29 | 0.772 | 0.001 | 0.000 | 0.055 | 0.032 | 0.051 | 0.030 |
| 30 | 0.774 | 0.002 | -0.008 | 0.056 | 0.031 | 0.053 | 0.030 |
| 31 | 0.774 | -0.001 | -0.011 | 0.057 | 0.031 | 0.054 | 0.031 |
| 32 | 0.778 | 0.000 | -0.004 | 0.051 | 0.030 | 0.049 | 0.029 |
| 33 | 0.778 | 0.001 | -0.001 | 0.051 | 0.031 | 0.048 | 0.029 |
| 34 | 0.783 | 0.004 | 0.002 | 0.049 | 0.031 | 0.045 | 0.029 |
| 35 | 0.799 | 0.004 | -0.009 | 0.049 | 0.029 | 0.046 | 0.028 |
| 36 | 0.800 | 0.001 | -0.019 | 0.050 | 0.028 | 0.048 | 0.033 |
| 37 | 0.800 | 0.000 | -0.012 | 0.051 | 0.030 | 0.048 | 0.031 |
| 38 | 0.805 | 0.000 | -0.015 | 0.050 | 0.030 | 0.047 | 0.032 |
| 39 | 0.809 | 0.003 | -0.029 | 0.054 | 0.030 | 0.053 | 0.041 |
| 40 | 0.810 | 0.000 | -0.017 | 0.048 | 0.029 | 0.045 | 0.032 |
|  |  |  |  |  |  |  |  |
| Average | 0.746 | 0.001 | 0.006 | 0.058 | 0.033 | 0.055 | 0.035 |

Table S6 Simulation characteristics (2,000 replications) of c-index and c-mbc across 40 centers of 200 patients when the calibration slopes were not normally distributed.

|  | True | Bias | | SD | | rmse | |
| --- | --- | --- | --- | --- | --- | --- | --- |
| Cluster | concordance | c-index | c-mbc | c-index | c-mbc | c-index | c-mbc |
| 1 | 0.685 | 0.000 | 0.036 | 0.054 | 0.026 | 0.053 | 0.044 |
| 2 | 0.695 | 0.000 | 0.032 | 0.049 | 0.026 | 0.048 | 0.041 |
| 3 | 0.697 | 0.000 | 0.032 | 0.048 | 0.026 | 0.048 | 0.040 |
| 4 | 0.698 | 0.001 | 0.029 | 0.052 | 0.025 | 0.051 | 0.037 |
| 5 | 0.701 | 0.001 | 0.029 | 0.049 | 0.026 | 0.048 | 0.038 |
| 6 | 0.702 | -0.001 | 0.020 | 0.062 | 0.026 | 0.061 | 0.032 |
| 7 | 0.703 | 0.000 | 0.024 | 0.054 | 0.025 | 0.053 | 0.034 |
| 8 | 0.704 | -0.001 | 0.021 | 0.057 | 0.024 | 0.056 | 0.031 |
| 9 | 0.707 | 0.000 | 0.024 | 0.049 | 0.024 | 0.049 | 0.032 |
| 10 | 0.709 | 0.000 | 0.020 | 0.054 | 0.024 | 0.053 | 0.030 |
| 11 | 0.710 | 0.001 | 0.020 | 0.053 | 0.024 | 0.052 | 0.030 |
| 12 | 0.710 | -0.001 | 0.017 | 0.057 | 0.024 | 0.056 | 0.028 |
| 13 | 0.712 | 0.001 | 0.024 | 0.047 | 0.026 | 0.046 | 0.034 |
| 14 | 0.713 | -0.001 | 0.021 | 0.049 | 0.024 | 0.048 | 0.030 |
| 15 | 0.714 | 0.000 | 0.019 | 0.050 | 0.024 | 0.049 | 0.029 |
| 16 | 0.715 | 0.000 | 0.018 | 0.049 | 0.023 | 0.049 | 0.028 |
| 17 | 0.720 | 0.002 | 0.018 | 0.045 | 0.023 | 0.045 | 0.028 |
| 18 | 0.722 | 0.001 | 0.014 | 0.051 | 0.023 | 0.050 | 0.025 |
| 19 | 0.723 | 0.001 | 0.014 | 0.050 | 0.023 | 0.049 | 0.025 |
| 20 | 0.723 | 0.000 | 0.012 | 0.051 | 0.023 | 0.050 | 0.024 |
| 21 | 0.766 | 0.001 | -0.013 | 0.046 | 0.022 | 0.045 | 0.023 |
| 22 | 0.770 | 0.000 | -0.013 | 0.043 | 0.022 | 0.042 | 0.023 |
| 23 | 0.771 | 0.001 | -0.014 | 0.044 | 0.022 | 0.042 | 0.023 |
| 24 | 0.772 | 0.000 | -0.017 | 0.046 | 0.022 | 0.045 | 0.025 |
| 25 | 0.773 | 0.001 | -0.014 | 0.044 | 0.022 | 0.042 | 0.023 |
| 26 | 0.775 | 0.001 | -0.020 | 0.047 | 0.022 | 0.046 | 0.028 |
| 27 | 0.777 | 0.000 | -0.019 | 0.045 | 0.022 | 0.044 | 0.027 |
| 28 | 0.778 | 0.000 | -0.019 | 0.044 | 0.022 | 0.042 | 0.026 |
| 29 | 0.778 | 0.000 | -0.025 | 0.050 | 0.023 | 0.048 | 0.032 |
| 30 | 0.779 | 0.000 | -0.017 | 0.042 | 0.022 | 0.040 | 0.025 |
| 31 | 0.779 | -0.001 | -0.016 | 0.041 | 0.022 | 0.040 | 0.025 |
| 32 | 0.780 | 0.001 | -0.021 | 0.045 | 0.023 | 0.044 | 0.029 |
| 33 | 0.780 | 0.000 | -0.014 | 0.039 | 0.023 | 0.038 | 0.025 |
| 34 | 0.781 | 0.000 | -0.021 | 0.043 | 0.021 | 0.042 | 0.028 |
| 35 | 0.785 | -0.001 | -0.026 | 0.045 | 0.022 | 0.043 | 0.032 |
| 36 | 0.785 | -0.002 | -0.026 | 0.046 | 0.022 | 0.045 | 0.032 |
| 37 | 0.787 | -0.001 | -0.021 | 0.041 | 0.023 | 0.040 | 0.029 |
| 38 | 0.791 | 0.001 | -0.029 | 0.044 | 0.022 | 0.043 | 0.035 |
| 39 | 0.792 | 0.001 | -0.035 | 0.047 | 0.023 | 0.046 | 0.040 |
| 40 | 0.794 | 0.001 | -0.028 | 0.041 | 0.022 | 0.040 | 0.034 |
|  |  |  |  |  |  |  |  |
| Average | 0.744 | 0.000 | 0.001 | 0.048 | 0.023 | 0.047 | 0.030 |
